# Designing a Workplace-Based Learning Environment for Learning Health Promotion: A Design-Based Research

**DOI:** 10.5334/pme.1203

**Published:** 2024-06-27

**Authors:** Myrthe J. M. Verhees, Anneke M. Landstra, Rik Engbers, Marjolein H. J. Van De Pol, Renske Huijbregts, Roos A. Van Meggelen, Wietske Kuijer-Siebelink, Roland F. J. M. Laan

**Affiliations:** 1 Radboudumc Health Academy, Radboud University Medical Center, Nijmegen, Netherlands; 2 Rijnstate, Arnhem, Netherlands; 3 Department of Primary and Community Care, Radboud University Medical Center, Nijmegen, Netherlands; 4 Radboud University, she was an intern at Radboudumc Health Academy, Radboud University Medical Center, Nijmegen, Netherlands; 5 HAN University of Applied Sciences, Nijmegen, Netherlands

## Abstract

**Introduction:**

The healthcare landscape has a growing emphasis on health promotion (HP), which makes HP important in the training of future physicians. This study employed design-based research to develop a clerkship focused on HP and to outline design principles for shaping workplace learning environments to promote HP learning.

**Methods:**

We evaluated a nursing-home clerkship designed at Radboud University Medical Center in the Netherlands, and refined it over three rounds. Data collection involved individual and group interviews with students and supervisors, as well as observations during clerkship-related meetings and activities. These interactions also facilitated the exchange of perspectives between participants and generation of new design ideas, fostering co-creation of the clerkship design. Data were analyzed through iterative thematic inquiry to inform new design choices and develop design principles.

**Results:**

Evolved clerkship designs included an app for capturing practice experiences to discuss in relation to students’ professional roles, loosening the strict assessment structure, and collaborative creation of a practice assignment about ‘Positive Health’. We constructed four design principles, including: to question and discuss students’ professional identity, provide concrete and meaningful assignments, aim for a peer-learner role for supervisors, and foster co-creation of the workplace learning environment.

**Discussion:**

Our design principles support the design of workplace-based learning for HP, a subject that is novel within healthcare practice. We find that co-creation of workplace-based learning, which requires embracing uncertainty, is pivotal in this context, for students, practitioners, and educational institutions.

## Introduction

The healthcare landscape is undergoing significant changes, including a growing emphasis on health promotion (HP) and disease prevention. Promoting health and preventing disease is becoming increasingly important as the burden caused by lifestyle-related chronic diseases continues to rise [1,2]. Empowering and supporting individuals towards their individual health promotion also aligns with the current paradigm shift towards patient-centered care [3]. Alongside other healthcare professionals, physicians play a vital role in promoting health, which makes HP an important subject in the training of future physicians [4,5].

When it comes to learning about HP in medical education, workplace-based learning plays a crucial role. HP entails understanding each patient as a unique individual with specific health needs and a unique personal context. Learning about HP goes beyond the acquisition of knowledge and skills; it entails a shift in one’s perspective on healthcare and a change in attitude [6,7]. This learning requires active engagement with patients and fellow healthcare professionals in authentic practice settings: classroom-based learning may be less effective in fostering the needed transformative perspective change and application in practice [8,9,10,11].

Nevertheless, a comprehensive grasp of the ideal approach for structuring workplace-based learning about HP still eludes us. Literature on health advocacy, the CanMEDS role that includes HP, highlights several challenges in this regard. Health advocacy as well as HP are currently still finding their place in healthcare practice, and practitioners are exploring how these themes align with their professional roles [12,13]. This presents a difficulty in practitioners’ ability to serve as role models for students [14]. Because the theme of HP is still less pronounced than other curriculum themes and is often poorly defined in terms of assessment, this leads to students attaching less importance to it [14,15,16,17]. Due to these challenges, existing literature on workplace-based learning offers limited guidance for designing workplace-based learning focused on HP. For instance, while literature on professional identity formation and communities of practice centers learning from role models and increasingly participating in and adapting to established practices [18,19], HP role models and practices are often not readily available.

We aim to provide insight into challenges and considerations associated with HP learning within medical curricula. We adopt a design-based research methodology, actively involving practitioners and students, to answer the research question: how can a workplace-based learning environment be designed to benefit learning about HP? This paper outlines the design process of a nursing-home clerkship around the topic of HP, and presents design principles to guide the design of workplace learning environments to promote HP learning.

## Methods

### Context: the pilot-clerkship ‘Generalist Care and Health Promotion’

This study was conducted in a pilot clerkship titled ‘Generalist Care and Health Promotion’ (hereafter referred to as the clerkship) at the Radboud University Medical Center (Radboudumc) in the Netherlands. Two general practitioner faculty educators from the Department of Primary and Community care, one of whom specialized in elderly care, developed and coordinated the clerkship. Medical students who had (nearly) finished their first three years of medical school were able to voluntarily sign up for this clerkship, which preceded their regular clerkships. The clerkship aimed to give students early exposure to generalist care and HP outside of the hospital setting. HP was operationalized as HP in the context of individual patient care, and focused on various elements: nutrition, physical activity, goal-setting, sleep, relaxation, and social environment.

The clerkship consisted of a preparatory course of one week followed by a seven-week clerkship in a nursing home. The decision to opt for nursing homes as settings for the clerkship was driven by the opportunity to cultivate innovation within a small-scale healthcare environment, departing from more traditional hospital clerkships, and aligning with the aim of introducing students to extramural care for the elderly early in their training. Moreover, the nursing home context offered a compelling environment for HP learning, given the complexity of healthcare needs among the population, the longitudinal element in care, and the potential for interprofessional collaboration. Students were linked to a daily supervisor, a physician not in medical specialist training. Weekly peer-consultation meetings (PCMs) were guided by the general practitioner faculty educators. During PCMs, clerkship experiences and assignments were discussed. Practice based assignments guided students’ experiences and were designed around HP-related topics. Assessment involved students collecting feedback on their actions in clinical practice, using forms derived from the mini clinical evaluation exercise [20], working towards Entrustable Professional Activities [21]. Based on this, combined with a written reflection report, students were given a pass or fail grade.

### Study design

We undertook a design-based research (DBR), an emerging method for educational research, seeking to increase the impact, transfer, and translation of education research into practice [22]. In DBR, researchers and practitioners work together to design and develop feasible educational activities by studying consecutive versions of these activities in their contexts, while reflecting on the process to produce design principles that can guide practice [23]. For our study, this meant we worked together with learners, supervisors, and coordinators (see below, participants and the research team) to design and develop the clerkship. Our efforts were driven by the dual goals of DBR: contributing to theoretical understanding, in our case of workplace-based learning about HP, and developing interventions that are suitable in the context of the study, in our case to develop interventions that were contextually relevant and responsive to the needs of learners and educators in the clerkship.

**Figure 1:**
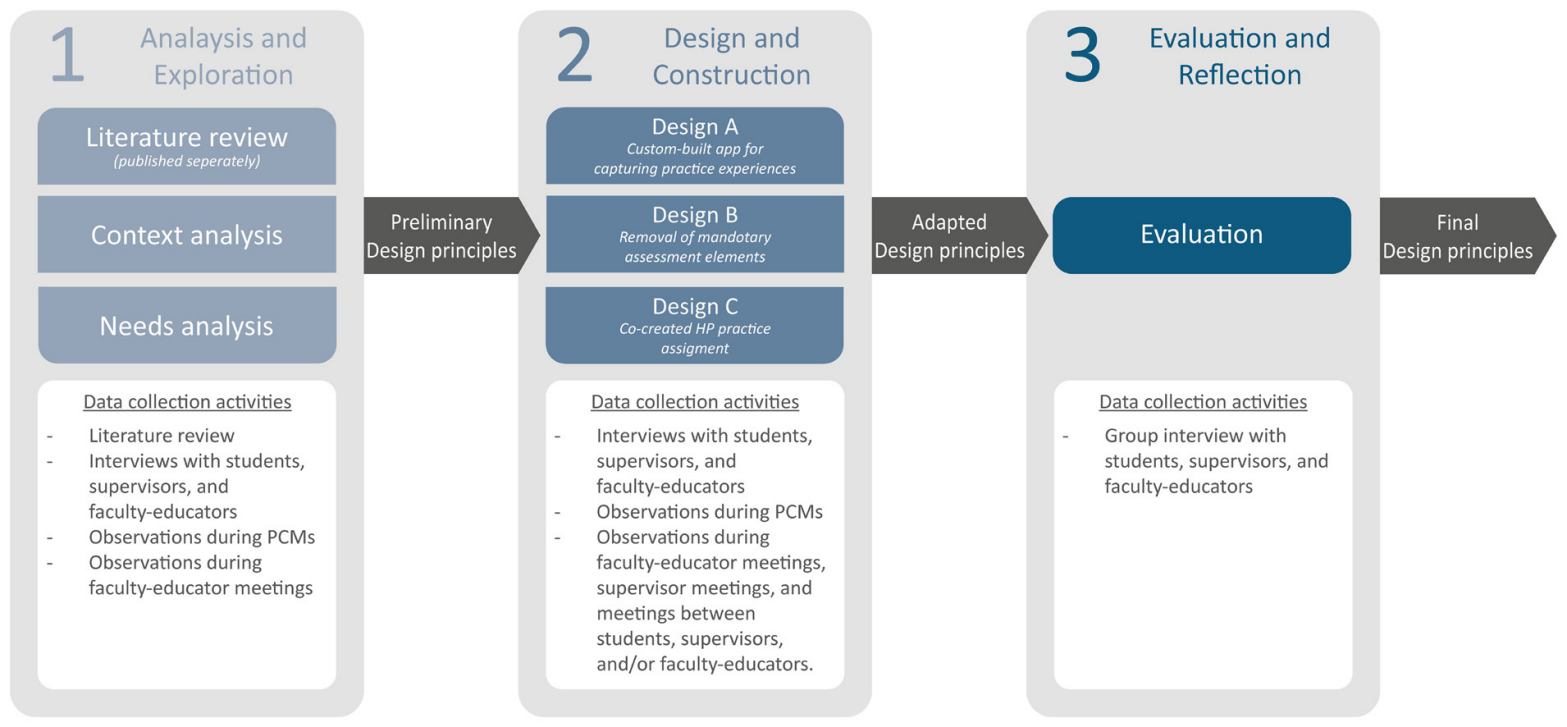
Different phases and associated data collection activities. Different phases of our Design-Based Research project, showing the activities in different phases of the study. The content of design A, B, and C is explained in more detail in the Results section.

DBR typically involves four phases: analysis and exploration, design and construction, evaluation and reflection, and implementation and spread [24]. We report on the first three phases in this study (Figure 1). Studying implementation and spread beyond our own study context (phase 4) was unfeasible given the established end date of the studied clerkship. As part of Phase 1, we conducted a scoping literature review [25]. The scoping review was focused on how healthcare professionals (in training) learn HP in the context of individual patient care through workplace-based learning. We evaluated included studies using a template based on the ‘designable elements of learning environments’ model [26]. We found that learning from authentic community-based healthcare practices was beneficial, as well as reflection on the health promoter role and addressing disparities between theory and practice. We observed challenges in clarifying health promotion within curricula and assessments, as well as in accessing role models due to the novelty of the topic in many practices. These findings informed our design choices and design principles.

### Participants and the research team

The primary researcher (MV) invited general practitioner faculty educators, students enrolled in the clerkship, and students’ supervisors to participate in the research. We emphasized that they were not just participants, but would be co-creators of the clerkship (see Data collection). To complement the expertise of the research team, we consulted different experts throughout the research. For the topic of HP, we consulted an elderly care physician in training, who specializes in HP and lifestyle medicine. The application (see Results) was built by two independent developers (see Acknowledgements).

The research team consisted of eight individuals with diverse backgrounds and expertise, to enrich reflexivity in the research process and enhance the analysis and design process. Their diverse range of expertise contributed to a comprehensive understanding of the multifaceted aspects of HP learning, encompassing clinical, educational, and institutional perspectives in data analysis and clerkship design. Participation of medical students RH and RM offered invaluable insights into the learner experience and perspectives, RH also participated in the first round of the clerkship. Involvement of MP and RL, who held leadership positions within medical institutions, provided insights into the organizational structures and policies influencing medical education. Educational experts RE and WK provided theoretical frameworks and methodological expertise to ensure the effective design and evaluation of the educational interventions. MV, AL, and MP provided a clinician perspective on challenges and opportunities for integrating health promotion into clinical practice.

### Data collection

Data collection occurred in between October 2021 and June 2023. We gathered data through formal data collection activities such as interviews and focus groups (Figure 1). Additionally, researchers were present at clerkship-related activities, such as PCMs and meetings related to the clerkship between supervisors or faculty educators. These moments included MV posing questions to stakeholders, facilitating exchanges of ideas between participant groups, and encouraging the sharing of diverse perspectives. MV also shared preliminary design principles, or findings from the literature review [25]. These interactions provided participants opportunities to share their perspectives, suggest design ideas, and provide feedback on proposed design elements and iterations. By closely aligning ourselves with and immersing ourselves in the participants’ practices, we aimed to foster co-creation of the clerkship and its design principles.

MV facilitated data collection activities and attended all relevant meetings, assisted by RE, RH, or RM. Data sources consisted of individual and group interviews with students, individual and group interviews with supervisors, observations of meetings and PCMs, and research team meetings. To evaluate design choices during the clerkships, we opted for individual interviews with students and supervisors, or separate group interviews with either students or supervisors, based on participants’ availability and time constraints. This approach aimed to create an environment for open dialogue, unhampered by any perceived dependency relationship. However, brainstorming new design ideas during PCMs or meetings, as well as the focus group in phase 3, were done with mixed groups of both students and supervisors to support co-creation.

Data were in the form of meeting minutes, audio-recorded interviews, and memos written by MV. Interview guides were constructed initially based on literature review and research team meetings, and were adapted as data collection proceeded (Appendix 1). Audio-recorded interviews were transcribed verbatim, and all data were anonymized prior to being shared with the research team. Due to the small scale of the research and the lack of suitable quantitative measures to evaluate HP (workplace-based) learning, we chose to only use qualitative measures. Different qualitative data sources were triangulated, for instance meeting minutes and memos created based on observations helped contextualize the data and capture nuances and dynamics that may not have been evident through other data collection methods such as interviews. These memos served as a form of researcher reflexivity, enabling us to track our evolving understanding of the research topic and identify emerging themes or patterns. Discussions of these themes or patterns during research team meetings have facilitated continuous refinement of the interview guide and the development and adjustment of design principles with sufficient nuance.

### Data analysis

Data collection and analysis proceeded iteratively. In Phase 1, we used an inductive coding process to explore the context and needs related to workplace-based learning about HP, and constructed themes. These themes, translated into initial design principles, informed development of designs in Phase 2. Data on evaluation of consecutive designs in Phase 2 and the evaluation in Phase 3 were coded using these initial design principles as codes. Data elements that did not fit with the initial design principles were subjected to open coding, allowing for the exploration of additional principles. Throughout the study, MV drew from literature on designing learning (environments) on the school-work boundary, Professional Identity Formation, and Communities of Practice to better understand the themes and to inform interview guides, designs, and revisions of design principles (e.g. [18,26,27]). In monthly research team meetings, facilitated and chaired by MV, open codes and memos were discussed to refine the design principles. Our approach, revising researcher-constructed themes throughout the research, aligns with iterative thematic inquiry [28].

### Ethical considerations

The Ethics Review Board of the Dutch Society for Medical Education granted ethical approval (case no. 2021.6.8). All individual participants gave written informed consent. Participation was voluntary. Students were explicitly told that their decision whether or not to participate would not influence their clerkship or assessment in any way.

## Results

The clerkship was offered four times, during which a total of 20 students, two faculty-educators, and 10 supervisors participated, all of whom participated in this study. The described results represent a synthesis of our findings across the different phases. We first provide a brief overview of the design choices we made, related to our scoping review findings. Elaboration on the development of these design choices, illustrated with stakeholder quotes, is provided under the description of the four design principles. The four design principles aimed at designing a workplace-based learning environment should be considered in conjunction to optimally benefit learning about HP.

### Designs of the clerkship

We worked together with participants to modify different elements of the clerkship based on our findings. We implemented three changes in the clerkship, each building upon the previous one.

Design A: We provided students with a custom-built app for capturing practice experiences. These experiences were then discussed during PCMs, with a particular focus on the experiences in relation to students’ ideas about their professional role. This approach was consistent with our literature review finding underlining the benefits of reflecting on health promotion practice.

Design B: In pursuit of creating space for workplace-based learning, we removed different mandatory elements imposed by the university. We removed the multiple HP practice assignments that were part of the default clerkship, as well as the requirement to collect a specific number of assessment forms. Our literature review finding that traditional assessment methods may not always align with HP learning substantiated this design choice.

Design C: We co-created a new HP practice assignment (Appendix 2) with students, supervisors, faculty-educators, and a HP expert. In this assignment, students talked to clients using the ‘Positive Health’ framework, and worked on a plan together with the client to improve the client’s health [29]. The choice of the Positive Health framework centers the needs and aspirations of clients, and helped students focus on the clients’ strengths, resources, and goals, encouraging a patient-centered approach to health promotion, and aligned with our literature review highlighting the need to make HP concrete for learners. The assignment suggested that students and supervisors learn from this assignment together: this was partly inspired by our literature review finding that opportunities for students and educators learning together were to date often overlooked in HP workplace-based learning.

The evaluation of these changes and their impact on HP workplace-based learning practice led us to construct the following four design principles.

### Design principle 1: Actively question students’ images of their future profession

To make students more open to relevant HP learning experiences, we found that it was crucial to engage them in discussions about the roles and responsibilities of physicians in HP in relation to their own experiences in practice. Students initially struggled to identify meaningful learning experiences in practice, due to a perceived mismatch between the expectations students had of their future profession, and the reality they experienced during the clerkship. Students felt that every learning experience had to be, as they said themselves, ‘medical’, and as they did not perceive HP as such, they overlooked HP learning experiences. As a student aptly said about this: “I did talk to clients about how they were doing, for example. But I’m not really sure … these weren’t actually medical conversations, so I found it difficult to relate them to my role as a doctor.”

We experienced that it was helpful to engage students in discussions about their notions of physicians’ roles and practices. We encouraged students to document practical experiences in an app, and to discuss those experiences with peers, faculty-educators, and supervisors (Design A). This idea was raised by three students, who expressed the need to capture fleeting moments throughout the day, which seemed not immediately ‘medically’ interesting or appeared to hold value for their learning, but may turn out to be relevant to look back on. Initially, they felt the app was a burden, an ‘extra’ thing to do. “Oh well, if this [app] is not being assessed, then I have enough different priorities that are being assessed.” Quotes such as this one, as well as observations shared during PCMs, highlighted students’ preoccupation with clerkship assessment, and underscored that learning in practice sometimes lagged behind the need to meet the various checkbox requirements. This prompted Design B. Once we made room for focus on practice experiences instead of assessment, students felt the opportunity to pay attention to their experiences in practice, including what amazed them or wanted to discuss again. This student describes how and why she used the app.

“Sometimes I marvel at something and then I think, oh, I’m going to jot that down in the app right away. And sometimes at the end of the day, on the train ride home, I reflect on like, what did I actually do today? I also just find it nice for myself to be able to read it back. And during PCMs, I thought, oh yes, I also experienced that. Otherwise, I would have completely forgotten about it, so in that respect, it’s really nice. It’s like a diary, kind of.”

During PCMs, students’ experiences were discussed, using questions like: ‘How did this experience differ from your initial expectations of practice?’ and ‘What aspects of this experience do you consider ‘not medical’?’ (Design A). Following these discussions, we noticed a shift in students’ perceptions of their future professional images, as they increasingly found practice situations to be instructive and relevant. This shift was evident in the quotes provided by two students, reflecting a heightened process of self-reflection in this particular domain.

“During the PCM, we talked about what kind of doctor we want to be. Over the past three years of the Bachelor’s program, this was not something I’d given much thought to. (…) I really enjoyed thinking about what matters to me.”

“I’m really a listener (…) and I’d like to be a doctor who really listens, up to a point, of course, because you don’t always have time to listen to people’s life stories…. Still, I’d like to be a doctor who really listens to people, instead of just focusing on the medical aspect of treatment. I’ve seen my supervisor do that too, really take a moment to sit down and have a chat with people.”

### Design principle 2: Provide concrete and meaningful practice assignments to support workplace-based learning

The initial assignments about HP were interpreted by students as very directive, and a mismatch was experienced between what they were supposed to do according to the assignment and the opportunities for this in practice. This hindered learning, and lead to removing them in Design B. As one student described: “I couldn’t complete the assignment because there was no multidisciplinary consultation this week… And the other assignments I also ended up doing mostly outside the clerkship.”

A collaboratively designed assignment (Design C) held value for HP learning for several reasons. The assignment was open-ended and flexible in nature, requiring collaboration with a patient and therefore anchoring the assignment in real life practice. The framework of Positive Health, suggested by an HP-expert and one of the clerkship supervisors, provided a tangible structure for approaching HP. According to students, the assignment provided a meaningful learning experience. Supervisors observed that it empowered students and added value in the practical setting. The quotes below reflect this.

Student: “About Positive Health… I enjoyed that it allowed me to immediately start talking to residents myself (…) and that I had to take a really broad perspective, not just looking at the disease, but also: What does this person enjoy doing? And is there anything I can change in this respect? Can I encourage someone to change their behavior? Stuff like that.”

Supervisor: “I sent an intern to talk to one of my patients about Positive Health. I felt this really added value because I don’t do it myself, or don’t pay enough attention to it. (…) What I liked was that the Positive Health approach really helped the intern to formulate a plan, in this case to help the resident give more meaning to their life.”

### Design principle 3: Aim for a peer-learner role for supervisors in the context of HP

The supervisors, as they themselves indicated and as echoed by students, were not heavily engaged in HP in their daily practice. This aspect challenged students to perceive them as HP role models. The Positive Health assignment included the suggestion that supervisors and students learn from it together (Design C). We saw that this shifted dynamics between student and supervisor, not only within the context of the assignment but also beyond it. The quotes below demonstrate how the collaborative development and execution of a practice assignment changed the way students and supervisors approached learning and working together.

Student: “It’s nice to be able to share my experiences with my supervisor. We often debrief together to reflect, for instance on my experiences with spiritual care. I’ve noticed that she appreciates hearing about my experiences; it’s like she’s learning something new too.”

Supervisor: “It certainly helped me too. Of course, it’s not about me, but… especially in difficult conversations, you don’t usually have anyone who is there to witness it (…), it is nice to get feedback on how calm you remain yourself in such a situation. You don’t usually get this kind of feedback in your daily practice (…) so it was really a mutual learning experience.”

### Design principle 4: Make room for co-creating the workplace-based learning environment

Our deliberate design choices as well as the participatory nature of our research methodology evoked discussions, sharing of perspectives, and the exchange of ideas between students, supervisors and faculty-educators to foster an understanding of what HP entails and how it can be integrated into practice. This fostered a sense of co-creation of the workplace-based learning environment. This supervisor described the impact of an open meeting between students, supervisors, and faculty-educators on the design of the clerkship:

“We discussed the design of the internship, as we sat together at the table, which is not something I’m used to. And we discussed interns’ learning objectives, really broad objectives, right? Not like teaching an abdominal examination… it allowed me and the student to tailor learning to the students’ personal learning goals, and to discuss, for instance, what is the essence of the care we deliver here?”

Throughout the study, we gradually let go of the predefined curriculum and assessment structure (Design B) to make room for shaping the learning environment collaboratively in practice. Initially, this led to uncertainty about how to proceed without the typical guidelines. The following quotes highlight the unease experienced by a student and a supervisor as they felt they had to comply with predefined expectations imposed by the curriculum:

Student: “I would have liked to have more structure, so that it would have been clearer beforehand what I had to do to pass. As in: if you do it this way, or that way, then that’s good as far as the University is concerned.”

Supervisor: “I think the internship offered a lot of room for our own interpretation of the assignments… I feel a bit more guidance would have maybe allowed us to feel a little freer to give it our own twist, together. We did schedule our own weekly follow-up meetings, and they have been really valuable I think, but still, the feeling of not being sure you are doing the right thing remains.”

Working towards a culture of co-creating and shaping the learning environment entailed letting go, embracing greater uncertainty, releasing preconceived notions about roles, and taking time for reflection. It involved being less dependent on and submissive to the curriculum or assessment, and instead, taking charge in shaping a practice to learn and work in together. Quotes from a student and a supervisor reflect how that brought a sense of tranquility, space, and autonomy.

Student: “I guess it’s because I don’t experience this pressure, as in: these are the requirements, and you have to meet them. It’s more about being curious and eager to learn, and just seeing what happens. That’s the thing I like most about it, and it also gives me a really calm feeling, which helps me not tense up. And then I can just get a lot more from it… because I have to be honest: I still didn’t get started on the assignments, but on the other hand, when I look at all the experiences I’ve written down, and everything I’ve jotted down over the past few weeks, I’ve really learned a lot…. And I really think: Wow! Because even without the assignments I’m already learning so much. And that really makes me feel at peace.”

Supervisor: “I really liked how it was more focused on experience. Especially compared to how it worked when I was an intern, when the focus was much more on assessment. So that switch, that makes it a little more relaxed. And with the practice assignment, I also noticed that the intern could really take control, and get some more responsibility.”

## Discussion

This study focused on workplace-based learning about HP in a nursing home clerkship. Through cycles of design, evaluation, and redesign, we co-created the clerkship to better facilitate HP learning. We translated our gained insights into design principles to guide the design of workplace-based learning environments to facilitate HP learning.

Fundamentally, our efforts revolved around developing workplace-oriented learning for a unique subject within the realm of healthcare practice. This contribution enhances the ongoing discussion on medical education for an evolving profession. Traditionally, workplace-based learning implied learning from role models, and of students gradually become part of a community of practice through legitimate peripheral participation [18]. However, HP was still evolving in this practice, and roles and responsibilities were still being defined. For new themes in healthcare practice, there is an opportunity to synchronize practice development with learning about these new themes. Socialization, an important concept in literature on professional identity and communities of practice, takes on a different meaning when learning involves not only observing traditional role models but also actively participating in shaping practice while learning. By expanding the ‘community’ to encompass students as legitimate participants, the process of shaping the ‘practice’ could become a collaborative learning journey that benefits professionals, students, patients, and practice itself [30]. We found that this does require embracing uncertainty of all involved.

For instance for students, discussing their professional identity and reflecting on their beliefs about their profession requires an open, responsive stance (design principle 1). Integration of HP in their professional role may prompt a re-evaluation of students’ existing expectations of their profession, and it may be challenging to sustain this integration when curriculum or supervisor focus suggests other topics are more urgent [31]. The significance of discussing HP role integration or interpretation is highlighted in more literature on HP or health advocacy [7,32,33]. We found that providing a concrete framework to work with in practice, such as Positive Health for HP learning, also helps in this regard. This is more broadly highlighted in literature, where various frameworks or tools are used to concretize HP in curricula and for students [33,34,35].

We know from literature that students, often accustomed to highly structured curricula in which assessment drives learning, may not easily assume responsibility in shaping their own learning, let alone in the process of co-creating workplace-based learning [36]. Our findings suggest that offering empowering assignments and altering student/supervisor role dynamics (design principles 2 and 3), alongside explicit opportunities to co-create (design principle 4), alleviated this challenge.

We found that curricular guidelines and assessment regulations also proved compelling for faculty-educators and supervisors, preventing them to optimally shape their educator role in co-creating workplace-based learning. This aligns with literature about the challenges of integrating the educational role into the physician role, describing that many doctors do not perceive themselves as teachers [37]. Our study, however, provided promising insight into the beautiful dynamics that can occur when space for learning and development is provided by clerkship coordinators, leading to ownership in co-creation of the clerkship between faculty educators, supervisors, students, and experts in the domain of HP. It would be interesting to see if these dynamics also manifest themselves on a larger scale of a curriculum, beyond this small flexible pilot setting.

The approach of DBR, creating meaningful interventions or practices in collaboration with the field, aligns well with how educational development for new themes could be approached. Co-creation, shared responsibility, and embracing uncertainty, are vital both to the research methodology and to educational development. The research methodology is therefore a strength of this study: actively engaging different stakeholders in our research enhanced the practicality and relevance of the design and design principles. Although the study involved a relatively small number of participants, this allowed for a comprehensive and in-depth trajectory. Possibly the voluntary participation of students might have introduced a selected group that could have impacted the study’s outcomes. A limitation of our study is the lack of patient involvement as stakeholders. While we made this decision thoughtfully, prioritizing the vulnerability of nursing home clients, future research could explore opportunities to engage patients in co-creation of workplace-based learning and in HP learning. Moreover, including multiple contexts would have strengthened the study. For future research, the application of our design principles in different healthcare settings and contexts could offer valuable insights.

## Data Accessibility Statement

The data that support the findings of this study are available from the corresponding author upon reasonable request.

## Additional Files

The additional files for this article can be found as follows:

10.5334/pme.1203.s1 Appendix 1. Positive Health assignment.

10.5334/pme.1203.s2 Appendix 2. Interview Guide.
