# Supplementary material for: Designing a Workplace-Based Learning Environment for Learning Health Promotion: A Design-Based Research
Source: Perspect Med Educ. 2024 Jun 27;13(1):357–67. doi: 10.5334/pme.1203 (PMC11212777; doi:10.5334/pme.1203)
Supplement: Appendix 1. — Positive Health assignment. [file pme-13-1-1203-s1.pdf]

Appendix 1: Positive Health assignment

Objective: You familiarize yourself with 'Positive Health.' You learn how to discuss positive health with patients/clients, and how to collaboratively create a plan with the patient/client and the healthcare team to maintain or improve their health.

Background: People are more than their illness or condition. Yet, this is usually what we focus on. All the attention goes to their symptoms and health problems, and how to solve them. Positive Health has chosen a different perspective. The emphasis is not on illness, but on the people themselves, on their resilience and on what it is that makes their lives meaningful (source: https://www.iph.nl/en/).

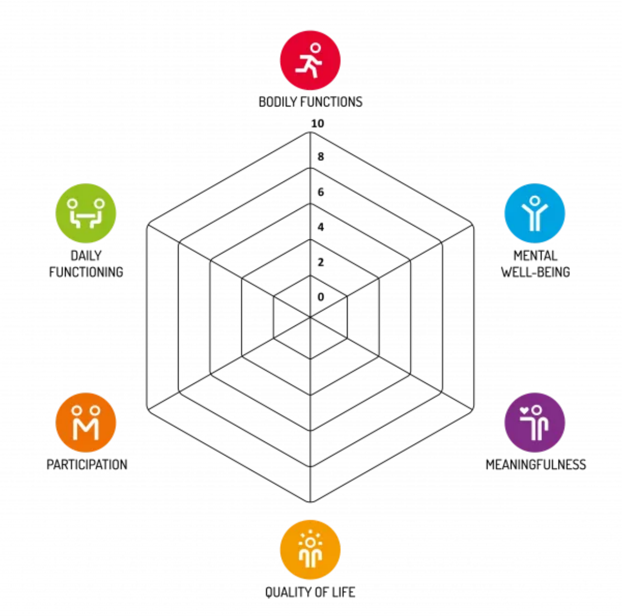

Image source: Institute for Positive Health (https://www.iph.nl/en/)

Your own Positive Health

1. Watch this video explaining what Positive Health means and how you can apply it in practice (https://www.iph.nl/en/knowledge-base/filling-out-the-spider-web-yourself/, Dutch video)
2. Complete the Positive Health questionnaire to gain insight into your perceived health. Afterward, ask yourself the following questions: What stands out? What do I find important? Is there something I would like to change? What could be a first step? (source: https://www.iph.nl/en/knowledge-base/filling-out-the-spider-web-yourself/)

Positive Health in practice

Positive Health is increasingly being applied in various medical fields, including general medicine, pediatrics, and geriatrics. Research conducted in a general practitioner's office demonstrated that it had a positive impact on patients' perceived quality of care and on the job satisfaction of healthcare workers. It also led to a reduction in referrals to secondary care and the number of prescribed medications. (Source (Dutch article): Peter Jung, H., Liebrand, S. & van Asten, C. *Uitkomsten van het hanteren van Positieve Gezondheid in de praktijk.* Bijblijven 35, 26–35 (2019). https://doi.org/10.1007/s12414-019-0075-x)

With the following assignment, you engage in Positive Health practice yourself. Work on this assignment together with your supervisor, allowing both of you to learn how to apply Positive Health.

1. Together with your supervisor, decide with which patient/client you want to have a conversation about their health, using the conceptual framework of Positive Health, and decide who will lead the conversation.
2. Complete the Positive Health questionnaire with the patient/client.
3. Afterward, discuss with the patient/client: What stands out to you? What do you consider important? Is there anything you would like to change? What could be a first step?
4. Collaborate with the patient/client to brainstorm and create an initial plan to implement changes. The medical student takes the lead in developing the plan and

coordinates with the supervisor. Identify and engage any other members of the healthcare team who can help.

Feel free to engage in these conversations with multiple patients/clients!

Discussion with your supervisor

Talk to your supervisor about this assignment. You can use the following example questions for your discussion, or think of questions yourself: What was it like to conduct the Positive Health conversation? What was the impact on the patient/client and their care? What insights will you take away from this experience to improve care for your future patients/clients? How do you envision its practical implementation? What aspects of Positive Health do you believe every doctor should be aware of?

Discussion during the peer consultation meetings

We will discuss the assignment during the peer consultation meetings.
