# Supplementary material for: Designing a Workplace-Based Learning Environment for Learning Health Promotion: A Design-Based Research
Source: Perspect Med Educ. 2024 Jun 27;13(1):357–67. doi: 10.5334/pme.1203 (PMC11212777; doi:10.5334/pme.1203)
Supplement: Appendix 2. — Interview Guide. [file pme-13-1-1203-s2.pdf]

Interview Guide – Students
Questions in italics were added in different iterations.

How did you experience the clerkship?

What have you learned during the clerkship? What have you learned about health promotion during the clerkship?

How do you view health promotion in relation to your future work as a doctor?

Can you describe a moment when you learned something meaningful or significant about this?

- What exactly did you learn? How did you learn about it?
- *What role did the supervisor play? What role did context of the nursing home play? What role did the assignment play? What role did assessment play?*

Thinking about how you learned about health promotion, what helped you? Or what could have helped you, what would benefit the next group?

How do you use the app? What benefits do you get from it? How could it help better/differently? What have you documented so far? Would you like to share about that?

How did you interact with you supervisor?

How was it for you to be involved in the design-based research study?

Interview Guide – Supervisors
Questions in italics were added in different iterations.

How did you experience the clerkship?

What do you think the medical student has learned? What has the medical student learned about health promotion?

What role did you play as a supervisor?

How did you interact with the medical student?

What did you find effective as a supervisor? What did you find challenging?

How did you work with the curriculum and with the assessment structures?

What did you gather from the assignments? From the preparation that medical students had? From the app the used?

What have you learned from the medical student?

How was it for you to be involved in the design-based research study?
